# Rapid Visual Detection of African Swine Fever Virus with a CRISPR/Cas12a Lateral Flow Strip Based on Structural Protein Gene D117L

**DOI:** 10.3390/ani13233712

**Published:** 2023-11-30

**Authors:** Desheng Zhang, Sen Jiang, Nengwen Xia, Youwen Zhang, Jiajia Zhang, Anjing Liu, Chenyang Zhang, Nanhua Chen, Francois Meurens, Wanglong Zheng, Jianzhong Zhu

**Affiliations:** 1 College Veterinary Medicine, Yangzhou University, Yangzhou 225009, Chinamx120200916@stu.yzu.edu.cn (Y.Z.); hnchen@yzu.edu.cn (N.C.); 2 Joint International Research Laboratory of Agriculture and Agri-Product Safety, Yangzhou 225009, China; 3 Comparative Medicine Research Institute, Yangzhou University, Yangzhou 225009, China; 4 Jiangsu Co-Innovation Center for Prevention and Control of Important Animal Infectious Diseases and Zoonoses, Yangzhou University, Yangzhou 225009, China; 5 Swine and Poultry Infectious Diseases Research Center, Faculty of Veterinary Medicine, University of Montreal, Saint-Hyacinthe, QC J2S 2M2, Canada; 6 Department of Veterinary Microbiology and Immunology, Western College of Veterinary Medicine, University of Saskatchewan, Saskatoon, SK S7N 5E2, Canada

**Keywords:** African swine fever virus (ASFV), D117L, Cas12a, lateral flow strip (LFS), sensitivity and specificity, detection

## Abstract

**Simple Summary:**

African swine fever, caused by African swine fever virus, seriously affects domestic pigs and wild boars and has brought huge economic losses to endemic countries and regions. However, there is still a lack of effective vaccines and therapeutics at present. The prevention and control of African swine fever largely depends on early and accurate detection, and thus the desired detection method of clinical African swine fever virus is indeed needed. Despite a number of detection methods available, we here develop a lateral flow strip detection method for African swine fever virus, based on the viral gene of structural protein named p17 and the new technology called the clustered regularly interspaced short palindromic repeats/Cas 12a nuclease system. This visual detection method is simple, rapid, sensitive, and specific and has the potential for clinical detection of African swine fever virus.

**Abstract:**

African swine fever virus (ASFV) is a large double-stranded DNA virus that is highly infectious and seriously affects domestic pigs and wild boars. African swine fever (ASF) has caused huge economic losses to endemic countries and regions. At present, there is still a lack of effective vaccines and therapeutics. Therefore, rapid and accurate detection is essential for the prevention and control of ASF. The portable DNA endonuclease (Cas12a)-mediated lateral flow strip detection method (Cas12a-LFS) combined with recombinant polymerase amplification (RPA) has been gradually recognized as effective for virus detection including ASFV. In this study, based on the ASFV structural protein p17 gene (D117L), an RPA-Cas12a-LFS detection method was established. The detection method exhibits a sensitivity of up to two gene copies and has no cross-reaction with nine other swine viruses. Thus, the method is highly sensitive and specific. In 68 clinical samples, the coincidence rate of the p17 strip was 100%, compared to the traditional quantitative PCR (qPCR). In conclusion, we have developed a simple, rapid, sensitive, and specific ASFV visual detection method and demonstrated the potential of on-site detection of ASFV.

## 1. Introduction

African swine fever (ASF) is an acute, febrile, and highly contagious infectious disease caused by African swine fever virus (ASFV), infecting domestic pigs and wild boars [1]. The acute type is characterized by high fever, depression, anorexia, skin cyanosis, splenomegaly, and bleeding, exhibiting extremely high morbidity and mortality [2]. ASFV is a 200 nm diameter icosahedral DNA virus and can be divided into 24 genotypes based on the major capsid protein p72 sequences [1]. In recent years, ASFV has been widely spread, with a huge impact on the global pig industry [3]. Since 2018, the outbreaks of ASF in China and several eastern Asian countries have significantly impacted the local swine industry, resulting in huge economic losses [4]. At present, there is still no effective vaccine against the disease, and there is no effective drug for treatment [5]. Therefore, early monitoring, diagnosis, and biosafety are very important for the prevention and control of the disease. Many methods have been developed to detect ASFV infections, including etiological and serological methods, such as conventional PCR, quantitative PCR, virus isolation, fluorescent antibody tests, and enzyme-linked immunosorbent assay (ELISA) [6,7]. However, these methods require expensive equipment, standardized laboratories, and/or professional technicians to perform them, and the time required for result availability is long, which is a problem for meeting the front-line requirements of pig farms [6,7]. Methods to detect ASFV more quickly, simply, accurately, and cheaply are the future development direction for ASFV infection management [6].

Compared to traditional pathogen antigen or antibody detection methods, nucleic acid detection technology is a powerful tool for early detection and diagnosis [8,9,10,11]. The clustered regularly interspaced short palindromic repeats (CRISPR)/Cas12a system recognizes matched double-stranded DNA (dsDNA) containing a protospacer adjacent motif (PAM) under the guidance of CRISPR RNA (crRNA) and then prompts the target dsDNA to unwind [12,13]. The target strand (TS) in the target dsDNA forms an Rloop with the crRNA after unwinding, thus releasing the active site of RuvC in Cas12a [12,13]. Next, the non-target strand (NTS) and TS of the target dsDNA will be sequentially cut in a cis way by the active RuvC [12,13]. At this time, once the single-stranded (ss) DNA enters the active site of RuvC, it will also be cut in a trans way. Through this way, the entered reporter ssDNA (probe) is subjected to cutting and acts as a readout for specific detection [14,15]. In addition, CRISPR/Cas12a was combined with recombinant enzyme polymerase amplification (RPA) and a lateral flow strip (LFS) to create a fast, accurate, sensitive, and specific detection platform [16,17,18]. The RPA-Cas12a-LFS method for detection of virus infections including ASFV has been widely recognized [19,20,21]. 

**Figure 1:**
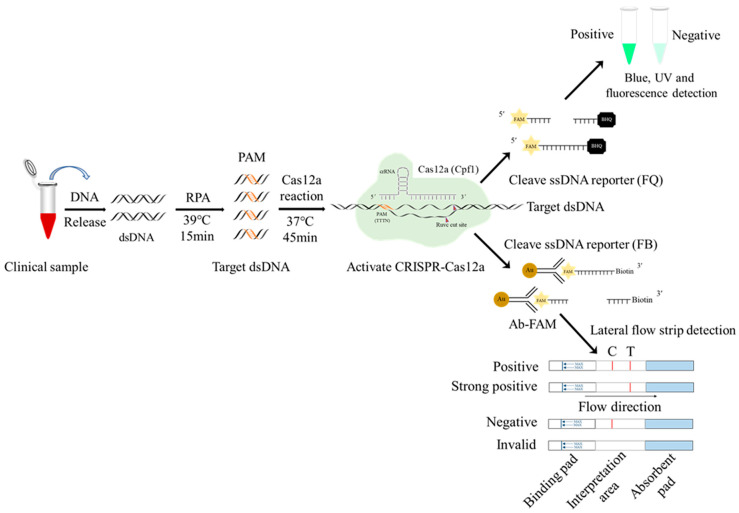
Schematic diagram of RPA-CRISPR/Cas12a-LFS detection of ASFV. The ssDNA probe labeled by 5′-gold-Ab-FAM and 3′-biotin is used for lateral flow strip (LFS) detection, whereas the ssDNA probe labeled by 5′-FAM and 3′-BHQ is used for blue light, UV detection, and fluorescence detection.

African swine fever virus (ASFV) encodes more than 160 proteins [22], and the viral capsid protein p17 is one of the major viral proteins. It is encoded by the conservative sequence D117L gene, which can be used as a target site for ASFV nucleic acid detection [23,24]. An indirect ELISA based on purified p17 was established and shown to effectively monitor the antibody levels in clinical ASFV infection [25]. This study developed RPA-Cas12a-LFS for ASFV detection based on the ASFV D117L gene (Figure 1), by designing the specific RPA primers, crRNA, and probe combinations, optimizing various CRISPR/Cas12a detection reaction parameters, and determining the sensitivity and specificity of the RPA-Cas12a-LFS detection method. Further, the D117L-RPA-Cas12a-LFS was applied to clinical sample detections, and the results showed complete consistency with conventional quantitative PCR assay. Therefore, this method can be used as an attractive alternative in clinical ASFV detection and provides an important tool for monitoring and controlling ASF. 

## 2. Materials and Methods

### 2.1. Reagents and Viruses

The plasmid 6His-MBP-TEV-huLbCpf1 was bought from Addgene (Cat #90096). The Rosetta (DE3) competent *E. coli* was obtained from Tiangen BioTech Co., Ltd. (Beijing, China). The His-tag Protein Purification Kit (p2226) was bought from Beyotime Biotech, Inc. (Shanghai, China). The FastPure^®^Gel DNA Extraction Mini Kit (DC301-01) and T7 High Yield RNA Transcription Kit (TR101-01) were both from Nanjing Vazyme Biotech Co., Ltd. (Nanjing, China). RNA Clean&Concentrator-5 (R1013) was purchased from Zymo Corporation (Irvine, CA, USA), while the recombinant polymerase amplification (RPA) kit (TwistAmp Basic, Cat #TABAS03KIT) was obtained from TwistDx Limited (Maidenhead, UK). The LbCas12a test strip (M20801-F007) was bought from Bio-Lifesci Company (Guangzhou, China). The Hipure Tissue DNA Mini Kit (D3121-02) and Hipure Blood DNA Mini Kit (D3111-02) were purchased from Magen Biotechnology Co., Ltd. (Guangzhou, China). The classical swine fever virus (CSFV) attenuated strain (CVCC AV1412) and porcine pseudorabies virus (PRV) attenuated strain (Bartha-K61) were from Jiangsu Nannong Hi Tech Co., Ltd. (Nanjing, China). The inactivated porcine parvovirus (PPV, S-1 strain) was purchased from Shangdong Huahong Biological Engineering Co., Ltd. (Binzhou, China). Other porcine viruses (PRRSV XJ17-5 strain, PEDV AJ1102-like strain, PKV SH-W-CHN-like strain, PCV2 JSYZ1901-660 strain, PCV3 JSNJ20-04-912 strain, and SIV H9N2 strain) were stored in our laboratory. 

### 2.2. Cloning of LbCas12a Gene and Protein Expression and Purification

The LbCas12a (*Lachnospiraceae bacterium* Cas12a) gene was amplified from the template plasmid 6His-MBP-TEV-huLbCpf1 by PCR using the pET28a-Cas12a-F/R primers (Table S1), and the PCR product was cloned into the *Xho* I/*Nde* I sites of the pET28a vector with ABclonal MultiF Seamless Assembly Mix (RM20523) to construct the pET28a-Cas12a expression vector (Figure S1A). The pET28a-Cas12a was transformed into Rosetta (DE3) competent *E. coli* and induced with 1 mM IPTG at 16 °C for 16 h to express the Cas12a protein. The expressed Cas12a protein was mainly present in the bacterial lysate supernatant (Figure S1B). The Cas12a protein from the lysate supernatant was purified at 4 °C with the His-tag Protein Purification Kit by several rounds of elution (Figure S1C). The eluted Cas12a protein was dialyzed in 600 mM sodium chloride, 5% glycerol, 2 mM DTT, 50 mM Tris HCl, pH7.5, for 3 times, 2 h each time, on a 4 °C shaker. The purified Cas12 protein was verified by SDS-PAGE plus Commassie blue staining and immunoblotting with anti-His antibody (Figure S1D,E). Our purified Cas12a protein has the same molecular weight and purity as commercial Cas12a protein (New England Biolabs, Ipswich, MA, USA) (Figure S1F).

### 2.3. Detection of Endonuclease Activity of the Purified LbCas12a Protein

To confirm whether the purified Cas12a protein has DNA cutting activity, the known target DNA sequence and crRNA were used to detect the protein’s activity [12]. The T1 site target DNA oligonucleotide chains (Table S1) were synthesized, and the T1 site target dsDNA was obtained by annealing. The crRNA-encoding DNA sequence was deduced as follows: T7 promoter (TAATACGATACTATAGG) + scaffold sequence of Cas12a (aatttctactaagtgtag) + target sequence (20–23 bp after target DNA PAM sequence). The complementary T1 crRNA-F and T1 crRNA-R (Table S1) were synthesized and annealed into double-stranded DNA, which was extracted by gel purification, and then transcribed in vitro using the T7 High Yield RNA Transcription Kit. The transcribed crRNA was purified using ZYMO RNA Clean&Concentrator-5 and verified by agarose gel electrophoresis. The ssDNA probe for Cas12a endonuclease, synthesized by Qingke Biotechnology Co., Ltd. (Beijing, China), is labeled with fluorescence group 6-FAM and quenching group BHQ-1 on the 5‘ and 3′ ends, respectively (5′-6-FAM-CCGGAAAAAAAAAAAACCGG-BHQ-3′). The ssDNA sequence in the middle of the Cas12a probe forms a hairpin structure, ensuring the high specificity of the probe.

The purified Cas12a protein was mixed with T1 target dsDNA, T1 crRNA, RNase inhibitor, and Cas12a probe in detection buffer (20 mM Tris HCl, 100 mM KCl, 6 mM MgCl_2_, 1 mM, DTT, 5% glycerin, 50 μg/mL heparin sodium, pH 8.0) in a brown RNase free centrifuge tube and subjected to reaction at 37 °C for 30 min. The Cas12a endonuclease activity was detected by blue light (wavelength 450 nm) and fluorescence signals (excitation wavelength 492 nm, emission wavelength 525 nm). The results of both blue light and fluorescence detection were consistently positive (Figure S2A,B), suggesting that the purified Cas12a protein has a good DNase activity.

### 2.4. Design of ASFV Structural Protein p17 Gene (D117L) crRNA and Its Mediated CRISPR/Cas12a Reaction

**Figure 2:**
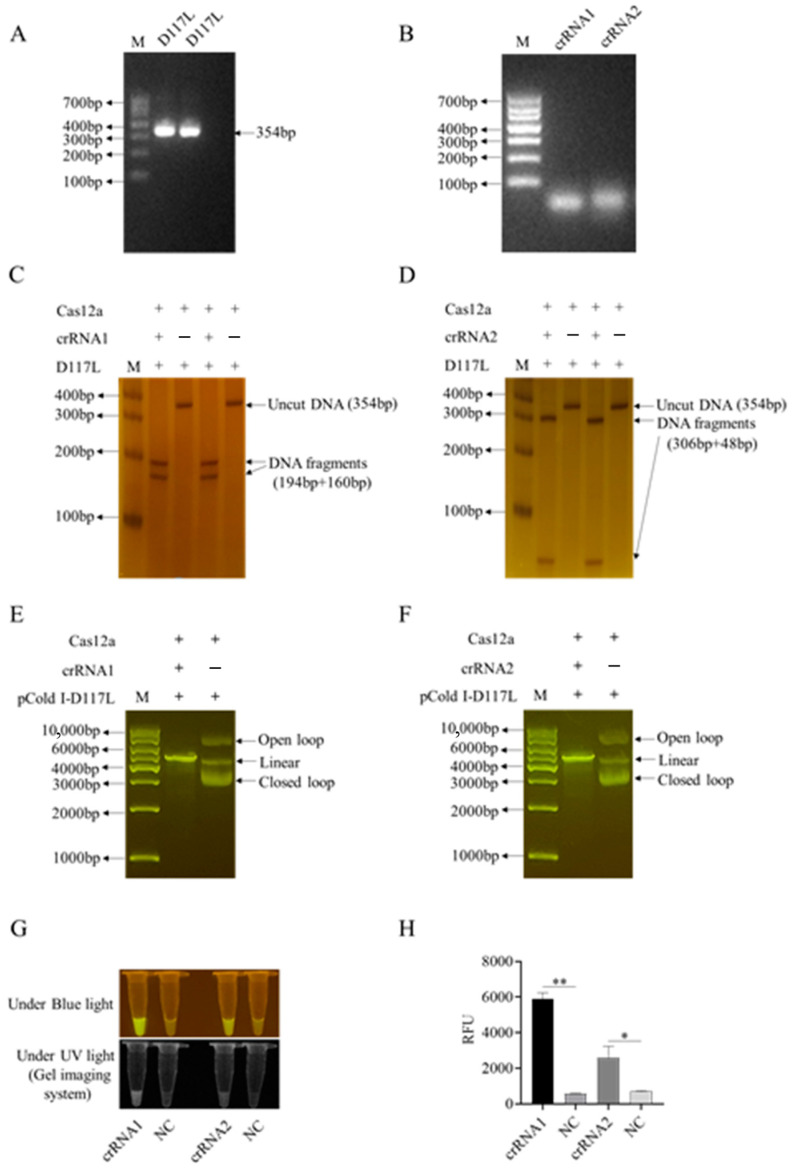
Two crRNAs of the ASFV D117L gene and the crRNA-mediated CRISPR/Cas12a reactions. (**A**) PCR amplification products of ASFV D117L gene in agarose gel. (**B**) In vitro transcribed D117L crRNA1 and crRNA2 in agarose gel. (**C**,**D**) Two-crRNA-mediated LbCas12a cleavage of D117L PCR products in non-denatured PAGE with silver staining. (**E**,**F**) Two-crRNA-mediated LbCas12a cleavages of pCold-I-D117L plasmids in agarose gels. (**G**) Detection of crRNA-LbCas12a reactions by blue light and ultraviolet light, respectively. (**H**) Detection of crRNA-LaCas12a reactions by fluorescence signal. NC denotes the reactions without template DNA. The Student’s *t* test; * *p* < 0.05 and ** *p* < 0.01 denote significant and very significant difference, respectively.

The D117L DNA sequence was based on the ASFV SY18 genome (GenBank No: MH766894), and the plasmid pCold-D117L constructed in our laboratory was used as the template DNA for PCR amplification. As shown in Figure 2A, the PCR fragment size of the full-length D117L coding sequence is 354 bp. The design, transcription, and purification of D117L crRNA referred to the known T1 target site crRNA. Two crRNAs targeting the D117L gene and one non-target crRNA were designed according to the special PAM sequence (TTTN) of Cas12a (Table S1) and were in vitro transcribed and purified. The generated two crRNAs were examined by gel electrophoresis (Figure 2B). The CRISPR/Cas12a reaction system was established as follows: 1 μL LbCas12a (250 ng/μL), 1 μL crRNA (150 ng/μL), 0.5 µL RNase inhibitor (40 U/μL), 44.5 μL detection buffer, 2 µL plasmid pCold-I-D117L (100 ng/μL) or 2 μL D117L PCR amplified fragment (110 ng/μL), and 1 μL ssDNA probe (10 μM), a total of 50 µL. The reaction mix was incubated at 37 °C for 20 min for LbCas12a-mediated cleavage to occur. The reaction products were verified by 1% agarose gel electrophoresis, blue light, UV light, and fluorescence detection, respectively.

### 2.5. Recombinant Polymerase Amplification (RPA)

According to the primer design principle of RPA, the optimal primer length is between 28 bp and 35 bp, and the optimal amplified fragment size is 150 bp. The RPA primers for the D117L gene were designed based on the same principle (Table S1). According to the TwistAmp Basic kit (TwistDx), each RPA reaction contains 29.5 μL buffer, reaction microsphere, 0.48 μM forward and reverse primers, 1 μL purified DNA template, 2.5 μL magnesium acetate (MgAc), and sterile water for a total volume of 50 μL. The RPA reaction was assembled in a clean environment and carried out under a constant temperature of 39 °C in a conventional water bath for 15 min. The amplified D117L gene products were observed by electrophoresis on 2% agarose gel and used for subsequent Cas12a lateral flow strip (LFS) detection. 

### 2.6. Establishment of RPA-CRISPR/Cas12a-Lateral Flow Strip (LFS) Detection Method

Firstly, the biotin-labeled ssDNA probe 5′-6-FAM-TTTTTTTATTTTTTT-Biotin-3′ was synthesized by Qingke Biotechnology Co., Ltd. Next, the gold particles cross-linked with the FAM antibody were combined with the above ssDNA probe to form a 5′-gold particle FAM antibody FAM-ssDNA-biotin-3′ strip detection probe. One tenth of the RPA product (5 μL), as the target DNA, was added into the CRISPR/Cas12a reaction system (1 μL LbCas12a, 1 μL D117L-crRNA1, 0.5 µL RNase inhibitor, 41.5 μL detection buffer, and 1 μL 20 nM gold particle biotin ssDNA probe), a total of 50 μL. After the reaction was performed at 37 °C for 20 min, the test strip arrow end of the binding pad was inserted into the CRISPR/Cas12a reaction solution. One to two minutes later, the strip identification area was completely soaked by liquid, and the quality control (C) line exhibited a gold line. Finally, the test results were read directly according to the presence of a gold line on the strip test (T) line (Figure 1). If the sample to be tested contains the ASFV genes, the CRISPR/Cas12a system cuts off the ssDNA probe and separates the FAM–gold particles from biotin. The biotin is captured by streptavidin on the quality control (C) line of the colloidal gold strip, while the FAM–gold particles continue to migrate forward and will be captured by the second antibody on the test (T) line. If the sample does not contain ASFV genes, the ssDNA report probe will be intact and will only be captured by the streptavidin C line of the colloidal gold strip (Figure 1).

### 2.7. Sensitivity and Specificity of D117L-RPA-Cas12a-LFS Detection Method

The plasmid pCold-I-D117L was 10-fold serially diluted where the plasmid concentration was from 2 × 10^10^ copies/µL to 2 × 10^0^ copies/µL. RPA amplification was performed on each plasmid dilution, followed by CRISPR/Cas12a-LFS detection. The minimum copy number of the target pCold-I-D117L plasmid was detected with test strips to determine their sensitivity. For specificity, viral DNA or RNA were extracted from the other nine swine RNA and DNA virus samples, including porcine reproductive and respiratory syndrome virus (PRRSV), porcine epidemic diarrhea virus (PEDV), porcine kobuvirus (PKV), classical swine fever virus (CSFV), swine influenza virus (SIV), porcine parvovirus (PPV), porcine pseudorabies virus (PRV), and porcine circovirus types 2 and 3 (PCV2/3). The above viral RNA or DNA were used as the target DNA to conduct the CRISPR/Cas12 reaction mediated by D117L crRNA1 followed by LFS detection to determine the reaction specificity of Cas12a-LFS detection.

### 2.8. Clinical Sample Processing and Nucleic Acid Extraction

The swine specimens, including 2, 4, 4, 6, 4, 2, 26, and 20 samples from heart, liver, spleen, lungs, kidneys, oral swabs, blood, and sera, respectively, were obtained and pro-vided by the Guangzhou Aisu Testing Technology Research Institute, Henan Branch. For tissue samples from the heart, liver, spleen, lungs, and kidneys, 0.2 g tissue each was mixed with 1 mL PBS and four steel balls in a 1.5 mL centrifuge tube. The tubes were then placed in an automatic grinder for homogenization with the set frequency 60 Hz, time 1 min, and centrifuged 12,000× *g* for 1 min. The oral swabs were eluted with 1 mL PBS, and the elution together with blood and serum samples were processed by centrifugation at 12,000× *g* for 1 min. The nucleic acids were extracted from 250 μL centrifugation supernatants by using the High Tissue DNA Mini Kit (D3121-02) and High Blood DNA Mini Kit (D3111-02) according to the instructions. The entire process took approximately 25 min.

## 3. Results 

### 3.1. CRISPR/Cas12a Reaction Mediated by ASFV Structural Protein Gene D117L crRNA1 and Its Optimization

First, we successfully established the D117L-crRNA-mediated CRISPR/LaCas12a reaction system. As shown in Figure 2C–F, two ASFV D117L crRNAs (crRNA1 and crRNA2) could both mediate Cas12a’s cutting of target dsDNA, including D117L PCR fragments (Figure 2C,D) as well as pCold-I-D117L plasmid DNA (Figure 2E,F). The CRISPR/Cas12a cutting of the DNA probe generated consistent and specific optical signals under the activation of blue light, ultraviolet light, or fluorescence (Figure 2G,H and Figure S2C,D). Next, we utilized the D117L crRNA1 in CRISPR reactions to optimize the pH, probe concentrations, and Cas12a/crRNA ratios by measuring the blue light, ultraviolet light, or fluorescence signals caused by LbCas12a cutting of the ssDNA probe.

The blue light, ultraviolet light, and fluorescence signals caused by the Cas12a cutting of the ssDNA probe were analyzed using detection buffers with different pH values. The results showed that when the pH value was 8.0, the light signal effects were better than at other pH values (Figure S3). With the increase of probe concentration from 25 nM, the signal intensity of the blue light, ultraviolet light and fluorescence signals increased continuously (Figure S4). Considering the balance of signal to noise, the probe concentration of 100–200 nM was considered as appropriate (Figure S4). The initial concentrations of Cas12a protein and crRNA1 were both set to 500 ng/μL, and the CRISPR/Cas12a reactions were carried out at different Cas12a/crRNA ratios (4:1, 2:1, 1:1, 1:2, and 1:4). Different Cas12a/crRNA ratios caused variations in Cas12a activity; the blue light, UV, and fluorescence signals of Cas12a were higher at a 4:1 Cas12a/crRNA ratio than at other ratios (Figure S5).

### 3.2. The Detection Sensitivity of the CRISPR/Cas12a Reaction 

The sensitivity of the CRISPR/Cas12a reaction system was directly determined by using 10-fold serially diluted pCold-I-D117L plasmids, with the plasmid concentrations from 2 × 10^10^ copies/µL to 2 × 10^0^ copies/µL. The results showed that the detection limit was 2 × 10^9^ copies/µL (Figure S6).

Further, the sensitivity of CRISRP/Cas12a combined with PCR was determined. PCR was first performed using 10-fold serially diluted pCold-I-D117L plasmids, and the PCR products were used for CRISPR/Cas12a reactions. The results showed that the detection limit could reach to 2 × 10^4^ copies/µL (Figure S7) and that early PCR amplification could significantly improve the sensitivity of CRISPR/Cas12a detection.

### 3.3. Establishment of CRISPR/Cas12a Mediated Lateral Flow Strip (LFS) Method, Its Sensitivity, and Its Specificity

**Figure 3:**
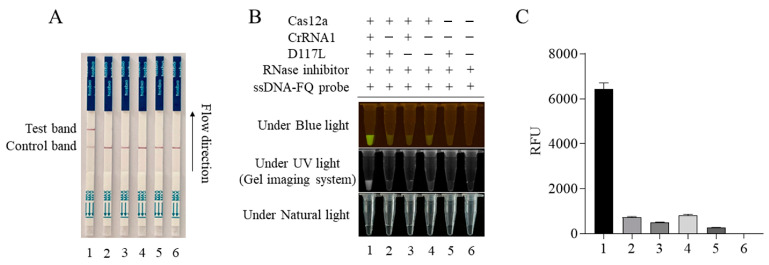
ASFV D117L-crRNA1-mediated CRISPR/Cas12a-LFS. The CRISPR/Cas12a reactions were set up in different samples of 1–6, as indicated in panel (**B**). The Cas12a reactions were subjected to lateral flow strip (LFS) detection (**A**) and also verified by blue light and UV light detection (**B**), as well as fluorescence detection (**C**).

The CRISPR/Cas12a-LFS detection method was established by combining the CRISPR/LbCas12a reaction with a lateral flow strip (LFS). The gold-labeled strip DNA probe (gold particles cross-linked with the FAM antibody plus 5′-6-FAM-ssDNA-Biotin-3′ DNA probe) was used. For negative samples, all gold-labeled DNA probes were intercepted by streptavidin at the quality control line (Line C). For positive samples, the gold- labeled DNA probe was cut by Cas12a; the gold-labeled anti-FAM antibody FAM complex at the 5′ end was broken and separated from the biotin at the 3′ end. When flowing through the quality control line (Line C) and the test line (Line T), respectively, the gold particles would be captured by the second antibody of Line T but decreased or disappeared at Line C. The results showed that only when Cas12a, crRNA, the template DNA, and the gold probe were all present would the gold particle signal appear on Line T of the test strip (Figure 3A). The Cas12a-LFS detection results were completely consistent with the blue light, ultraviolet light, and fluorescence signal results (Figure 3B,C).

**Figure 4:**
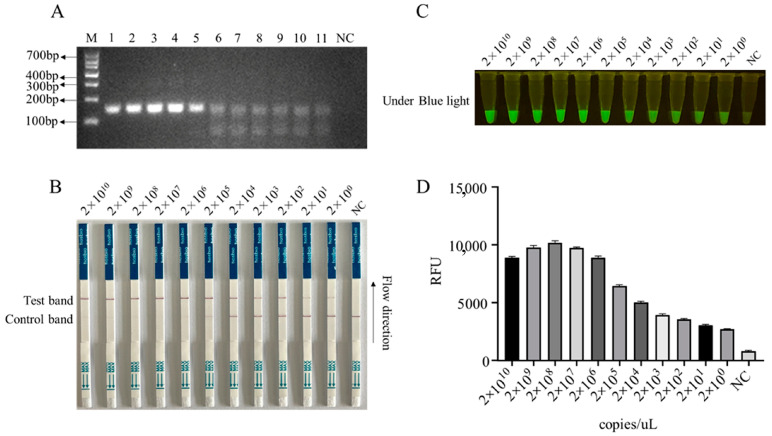
The sensitivity of RPA-CRISPR/Cas12a-LFS detection. (**A**) RPA products of D117L amplified from pCold-I-D117L with gradient concentrations from 2 × 10^10^ copies/uL to 2 × 10^0^ copies/uL (sample Nos. 1–11), with NC denoting negative control. The Cas12a-LFS detection of sensitivity was presented in (**B**) and also verified by blue light detection (**C**) and fluorescence detection (**D**).

**Figure 5:**
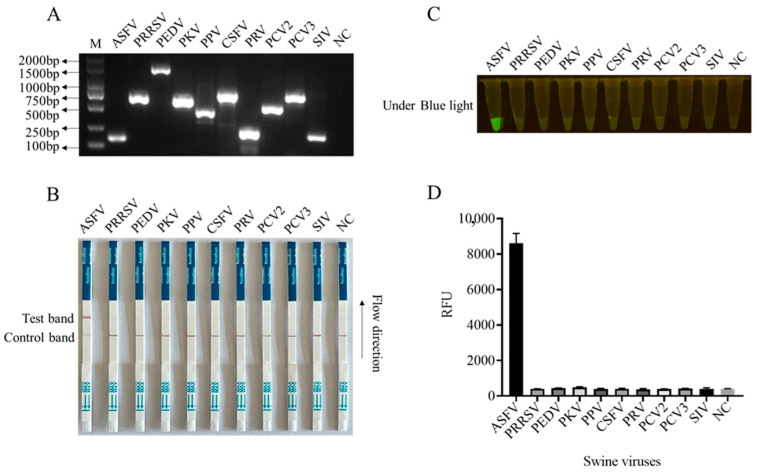
The specificity of CRISPR/Cas12a-LFS detection. (**A**) The specific gene PCR products of nine swine viruses as indicated were verified by 2% agarose gel electrophoresis. The NC denotes negative control. The specificity of Cas12a-LFS detection was presented in (**B**) and also verified by blue light detection (**C**) as well as fluorescence detection (**D**).

In order to enhance the sensitivity of the LFS detection method and ensure the efficiency and specificity of DNA amplification, we designed five pairs of RPA primers for the conserved sequence region of the ASFV structural protein p17 gene D117L (Table S1). Based on the specificity of RPA amplification products, the best RPA primers were screened (Figure 4A and Figure S8). In subsequent experiments, RPA primer pair 3 was selected for subsequent experiments (Figure 4A). Using 10-fold serially diluted pCold-I-D117L plasmids with concentrations ranging from 2 × 10^10^ copies/µL to 2 × 10^0^ copies/µL, RPA amplifications were performed, and the amplification products (Figure 4A) were applied for Cas12a-LFS detection. The results showed that the low detection limit of RPA-Cas12a-LFS could reach 2 × 10^0^ copies/µL (Figure 4B–D). For the specificity of the detection method, the nine porcine viral DNA or RNA samples were confirmed by regular PCR or RT-PCR (Figure 5A) and directly used for D117L-Cas12a-LFS detection. The results showed that all the T lines of the nine swine viruses were negative, and only the T line of the ASFV-positive sample showed gold particle positive (Figure 5B–D). Together, the results suggested that the RPA-Cas12a-LFS method has very high sensitivity and specificity for the detection of ASFV.

### 3.4. Detection of Clinical Samples by RPA-CRISPR/Cas12a-LFS

**Figure 6:**
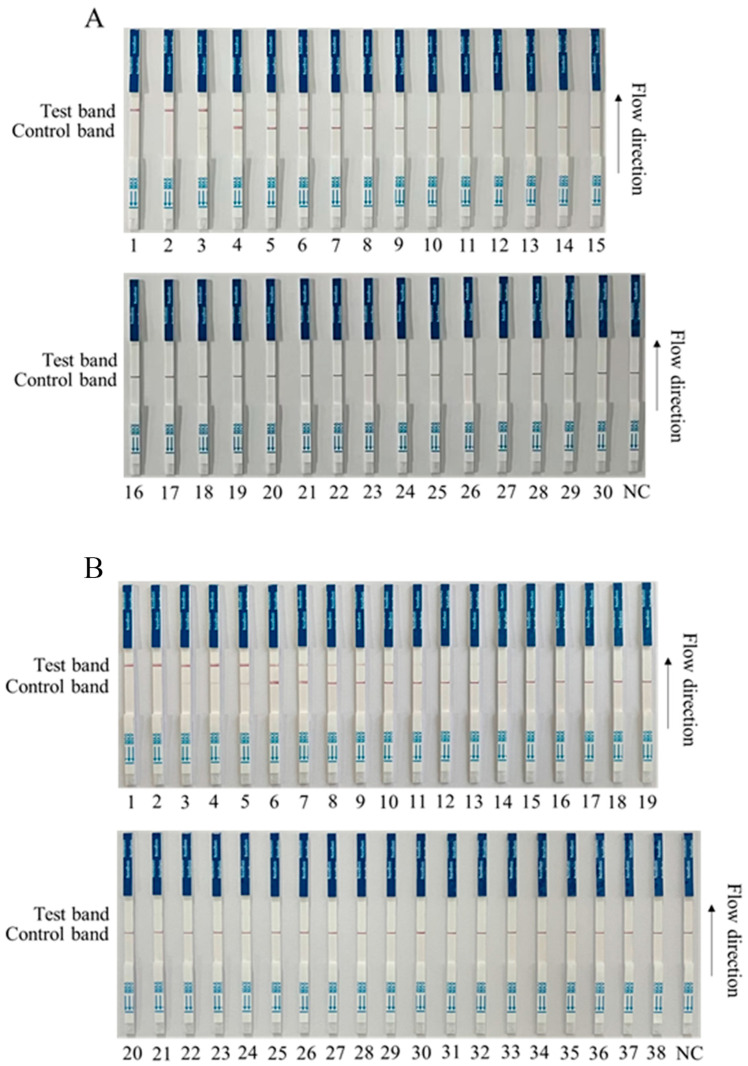
RPA-Cas12a-LFS detection of clinical samples. (**A**) Results of RPA-Cas12a-LFS detection of first batch of 30 clinical samples, with 8 positive (Nos. 1–8) and 22 negative (Nos. 9–30). (**B**) Results of RPA-Cas12a-LFS detection of second batch of 38 clinical samples, with 10 positive (Nos. 1–10), 3 weak positive (Nos. 11–13), and 25 negative (Nos. 14–38). The NC denotes the negative control.

We preliminarily tested for ASFV in a first batch of clinical samples (30 samples) by RPA-Cas12a-LFS, and the results showed that 8 of the 30 clinical samples were positive (Nos. 1–8), and 22 were negative (Nos. 9–30) (Figure 6A). Further, we tested a second batch of clinical samples (38 samples) with RPA-Cas12a-LFS detection for ASFV. The results showed that 10 out of 38 clinical samples were strongly positive (Nos. 1–10); three were weakly positive (Nos. 11–13); and 25 were negative (Nos. 14–38) (Figure 6B). In order to verify the reliability of the test strip for detecting ASFV, the quantitative PCR (qPCR) recommended by WOAH was used for verification detection [26]. The results showed that RPA-Cas12a-LFS detection was completely consistent with qPCR detection (Figure 6 and Table 1), indicating a 100% coincidence rate of the two methods and further confirming the reliability of the RPA-Cas12a-LFS detection method.

## 4. Discussion

In recent years, ASF has caused serious social and economic impacts, restricting the trade of pig products, and affecting food security [3]. Due to the lack of effective vaccines and treatments, the control strategy of ASF largely depends on the rapid detection of the infection and slaughter of infected pigs, which requires rapid, sensitive, and reliable diagnosis that can be applied to laboratory and field detection [6]. In order to cope with these huge challenges and help prevent the spread of ASFV, a better molecular detection method that meets the following standards is urgently needed to monitor and manage virus transmission [27,28]: (1) fast, providing diagnostic information before illness occurs; (2) simple, allowing unskilled personnel to use the detection method; (3) field applicable, which is very important for on-site ASFV testing. The RPA-Cas12a-LFS we developed is fast, simple, and has potential for field application. 

Until now, all the CRISPR/Cas detection assays have used ASFV p72 genes as the targets for detection [20,29,30,31,32], except one report that utilized the ASFV pp220 gene or DNA Pol gene as a detection target [33]. In this study, we selected a highly conserved ASFV structural protein p17 gene D117L sequence [23,24,34,35] as the detection target for CRISPR/Cas12a-LFS. We found that there are multiple PAM sequences (TTTN) in the conserved region of the D117L gene, and the designed crRNA based on PAM was able to mediate Cas12a reaction specifically and effectively. At the same time, we optimized the Cas12a reaction parameters, including pH, probe, and Cas12a/crRNA ratios, and achieved the visualization not only under blue light and ultraviolet light but also on a lateral flow strip (LFS). The detection sensitivity of our RPA-Cas12a-LFS can reach two copies, which is comparable to or better than the RPA-Cas12a-LFS based on the p72/B646L gene. From the virus nucleic acid extraction to the RPA-Cas12a-LFS reaction, it can be completed within 1 h and visualized with the naked eye, without need of expensive instruments and professional technicians. Further, the cost is about USD 0.61 for each detection, which is affordable. 

The RPA-Cas12a-LFS was able to differentiate strongly positive and weakly positive ASF samples. For negative samples, there will be only one gold band on the C line. For positive samples, the detection results depend on the efficiency of crRNA-mediated Cas12a cleavage. If the cutting efficiency of Cas12a is high in strongly positive samples, the ssDNA probe is completely cut; a high intense gold band will appear at the T line, and no band will appear at the C line, which was shown by several previous Cas12a-LFS studies [21,36,37]. In contrast, if the ssDNA probe is not completely cleaved by Cas12a in weakly positive samples, most of the gold band will appear at the C line, and only a faint gold band will occur at the T line, as we can see in Nos. 11–13 of the second batch of clinical samples.

In terms of detection specificity, we collected four kinds of swine DNA viruses and five kinds of swine RNA viruses for specificity analyses. Cas12a-LFS did not show any cross-positive reaction to other swine viruses, which proved that the detection system was highly specific and had important significance for clinical detection. In order to verify the reliability of RPA-Cas12a-LFS, 68 clinical samples were tested by RPA-Cas12a-LFS and verified by conventional qPCR. The results showed that the coincidence rate between the two methods was 100%. We conducted the D117L gene sequence alignments on different ASFV strains and found the D117L gene sequences are highly conserved across more than 100 ASFV strains of genotype II. Whether the D117L-LFS assay can detect other genotypes of ASFV is unknown. Due to the high variability of ASFV, we need to use more clinical samples that may contain different ASFV genotypes to evaluate the application value of our detection method in the future.

## 5. Conclusions

We have developed a rapid, sensitive, and specific RPA-CRISPR/Cas12a-LFS method for ASFV detection. It can be used for visual detection of ASFV in clinical samples and has potential significance for early monitoring, prevention, and control of ASF.

## Figures and Tables

**Table 1:** Comparison between RPA-Cas12a-LFS and qPCR detections of two batch clinical samples.

Sample Types	Sample Numbers	Results (Positive/Negative)	
		RPA-Cas12a-LFS	qPCR
Heart	2	1/1	1/1
Liver	4	2/2	2/2
Spleen	4	2/2	2/2
Lung	6	2/4	2/4
Kidney	4	2/2	2/2
Oral swab	2	0/2	0/2
Blood	26	9/17	9/17
Serum	20	3/17	3/17
Total	68	21/47	21/47
Positive rates		44.7%	44.7%

## Data Availability

Data are contained within the article and Supplementary Materials.

## Supplementary Materials

The following supporting information can be downloaded at https://www.mdpi.com/article/10.3390/ani13233712/s1. Figure S1. The expression and purification of the LbCas12a protein. Figure S2. Establishment of CRISPR/Cas12a reactions. Figure S3. Optimization of pH in D117L-crRNA-mediated CRISPR/Cas12 reactions. Figure S4. Exploration of probe concentrations in CRISPR/Cas12a reactions. Figure S5. Optimization of the Cas12a/crRNA ratios in CRISPR/Cas12a reactions. Figure S6. The sensitivity of D117L-crRNA-mediated CRISPR/Cas12a detection. Figure S7. The sensitivity of D117L-crRNA-mediated PCR–CRISPR/Cas12a detection. Figure S8. The RPA amplification results with different primers. Table S1: Primers used in this study.

## Abbreviations

ASFV, African swine fever virus; LFS, lateral flow strip; qPCR, quantitative PCR; ELISA, enzyme-linked immunosorbent assay; CRISPR, clustered regularly interspaced short palindromic repeats; crRNA, CRISPR RNA; PAM, protospacer adjacent motif; dsDNA, double-stranded DNA; ssDNA, single-stranded DNA; TS, target strand; NTS, non-target strand; RPA, recombinant enzyme polymerase amplification; CSFV, classical swine fever virus; PRV, pseudorabies virus; PPV, porcine parvovirus; PRRSV, porcine reproductive and respiratory syndrome virus; PEDV, porcine epidemic diarrhea virus; PKV, porcine kobuvirus; PCV, porcine circovirus; SIV, swine influenza virus.
