# Supplementary material for: Rapid Visual Detection of African Swine Fever Virus with a CRISPR/Cas12a Lateral Flow Strip Based on Structural Protein Gene D117L
Source: Animals (Basel). 2023 Nov 30;13(23):3712. doi: 10.3390/ani13233712 (PMC10705096; doi:10.3390/ani13233712)
Supplement: Supplementary file 1 [file animals-13-03712-s001.zip › Supplementary Figures.pdf]

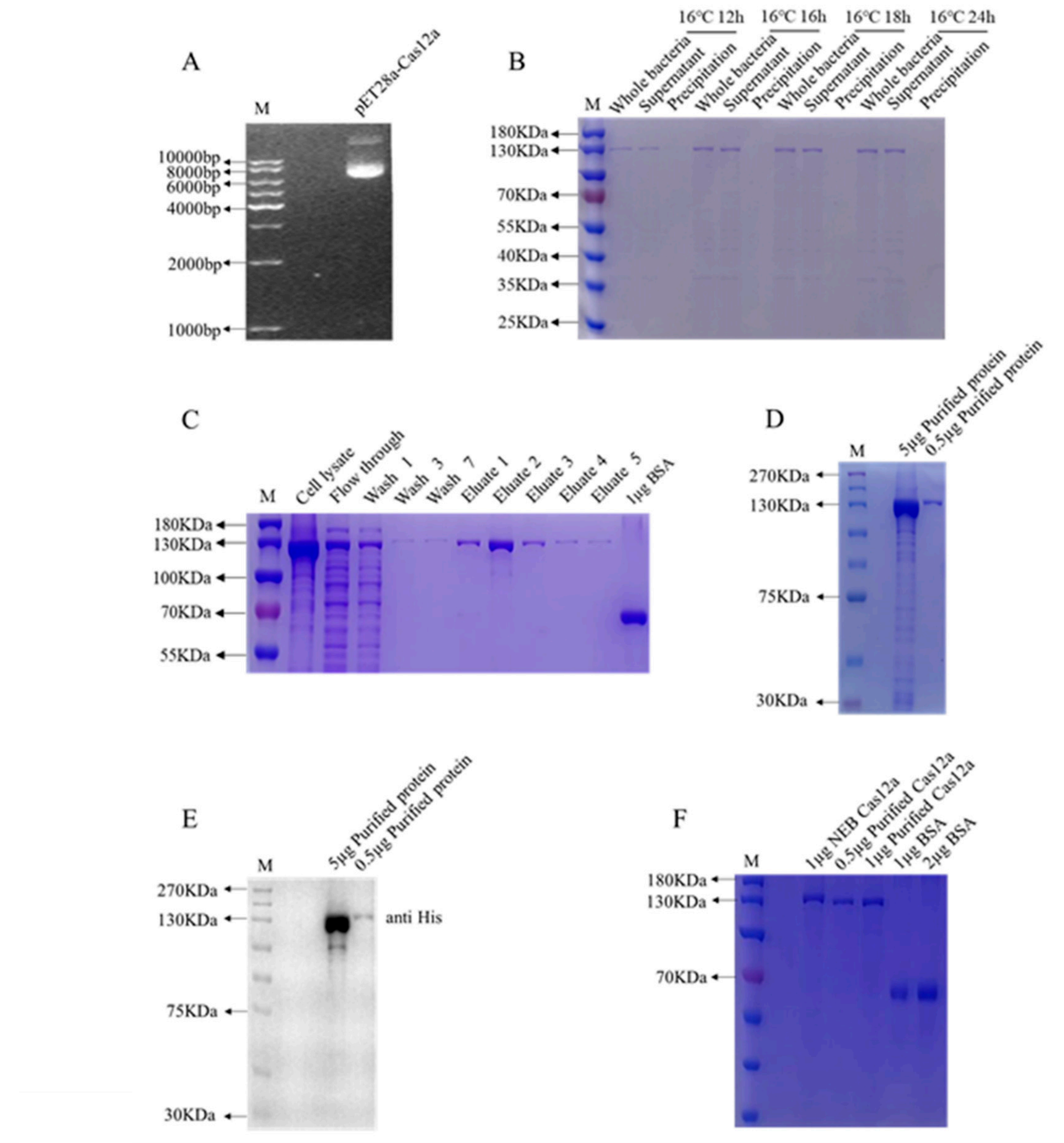

Figure S1. The expression and purification of LbCas12a protein. **A**, The recombinant pET28a-Cas12a expression plasmid. **B**, SDS-PAGE analysis of the Cas12a protein expressions in bacteria under different conditions. **C**, Purification of His labelled Cas12a protein by Ni^{2+}-NTA affinity chromatography. **D-E**, SDS-PAGE and Western blotting detections of purified Cas12a protein. **F**, Comparison between purified Cas12a protein and commercial Cas12a protein.

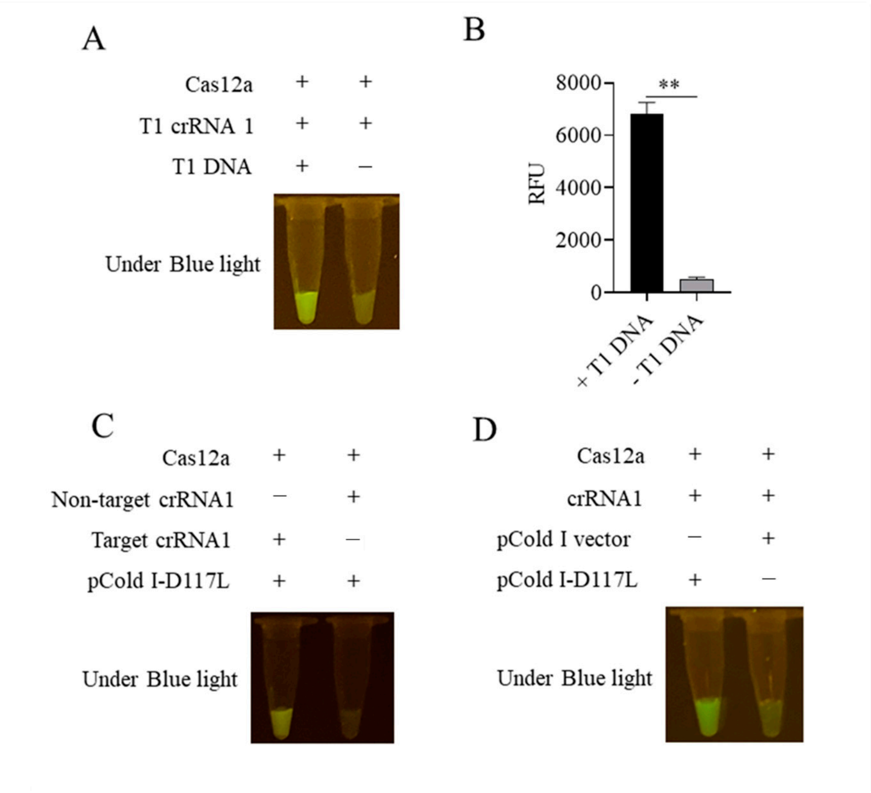

Figure S2. Establishment of CRISPR-Cas12a reactions. **A**-**B**, T1 crRNA1 mediated CRISPR-Cas12a reactions measured by blue light (**A**) and UV light (**B**). **C**-**D**, D117L crRNA or non-target crRNA1 plus pCold-I-D117L or pCold-I were combined for CRISPR-Cas12a reactions and measured by blue light.

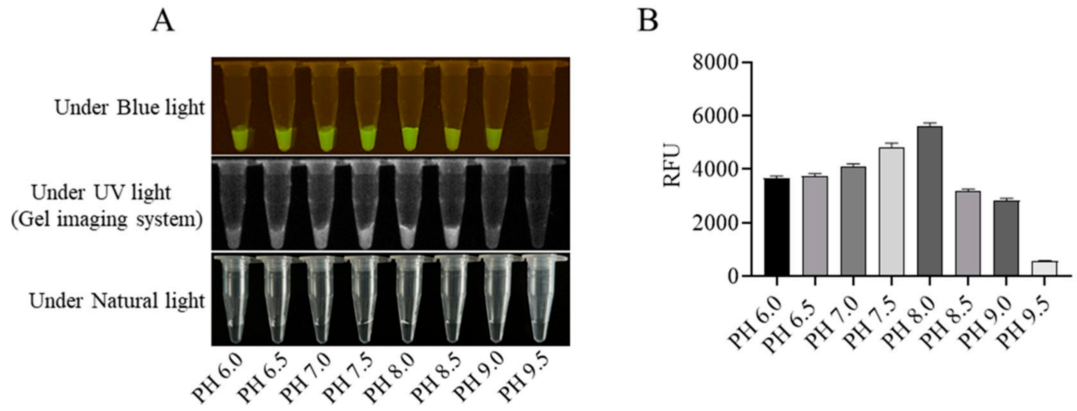

Figure S3. Optimization of pH in D117L crRNA mediated CRISPR-Cas12 reactions. The reactions with different pH values as indicated were detected under blue light and UV light (**A**), as well as by fluorescence (**B**).

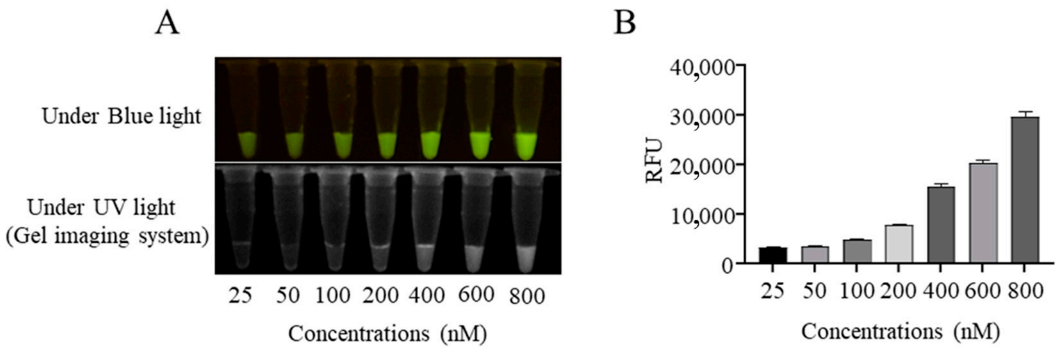

Figure S4. Exploration of probe concentrations in CRISPR-Cas12a reactions. The reactions with different probe concentrations as indicated were detected under blue light and UV light (**A**), as well as by fluorescence (**B**).

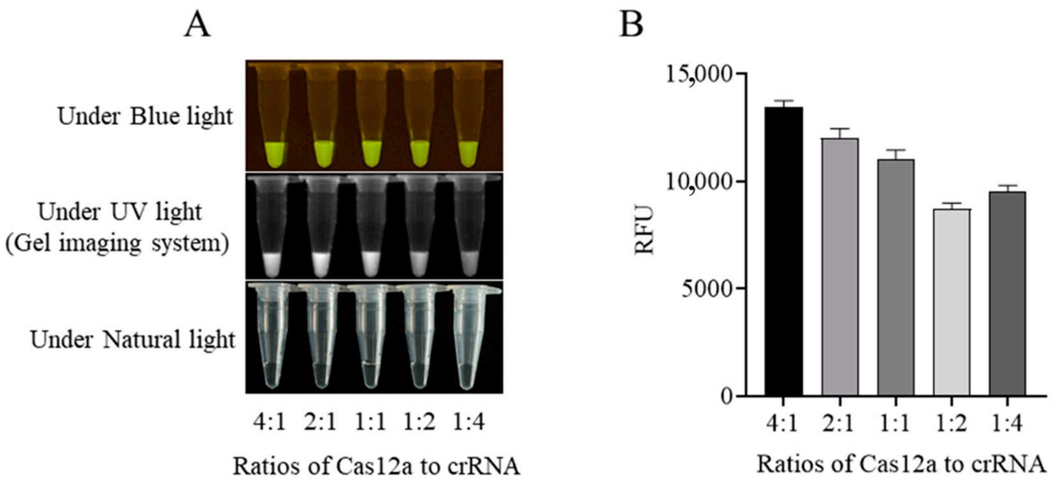

Figure S5. Optimization of the Cas12a/crRNA ratios in CRISPR-Cas12a reactions. The reactions with different Cas12a/crRNA ratios as indicated were detected under blue light and UV light (**A**), as well as by fluorescence (**B**).

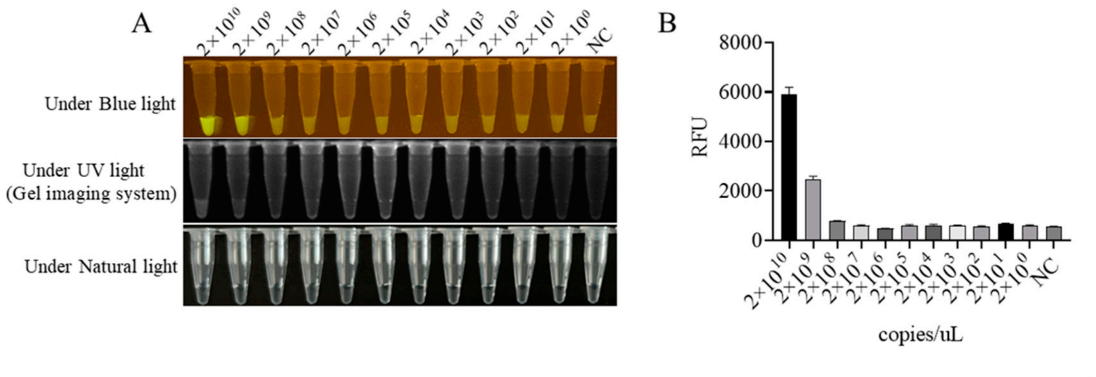

Figure S6. The sensitivity of D117L crRNA mediated CRISPR-Cas12a detection. The tenfold serially diluted pCold-I-D117L plasmid (from 2×10^{10} copies/uL to 2×10^{0} copies/uL) were used as target dsDNA for CRISPR-Cas12a reactions. The sensitivity of Cas12a detection was examined by blue light and ultraviolet light (**A**), as well as by fluorescence (**B**).

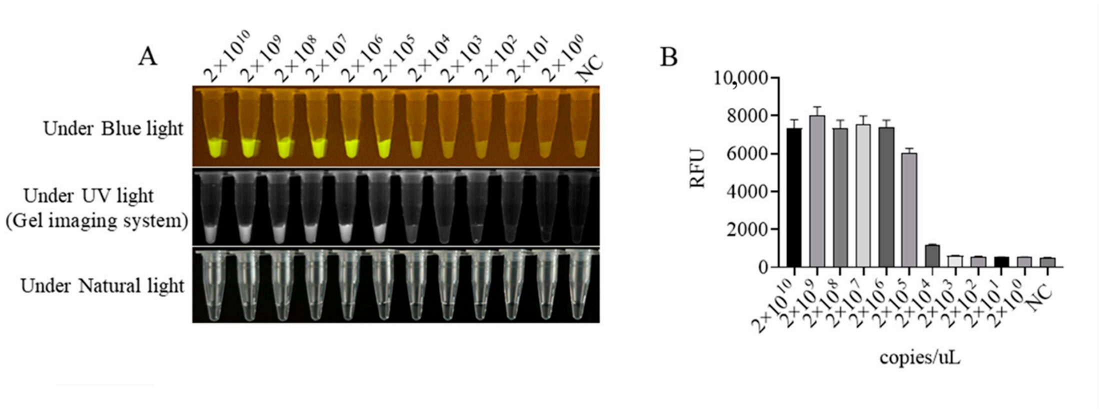

Figure S7. The sensitivity of D117L crRNA mediated PCR-CRISPR-Cas12a detection. The tenfold serially diluted pCold-I-D117L plasmid (from 2×10^{10} copies/uL to 2×10^{0} copies/uL) were first amplified by PCR, and the PCR products were used as target dsDNA for CRISPR-Cas12a reactions. The sensitivity of Cas12a detection was examined by blue light and ultraviolet light (**A**), as well as by fluorescence (**B**).

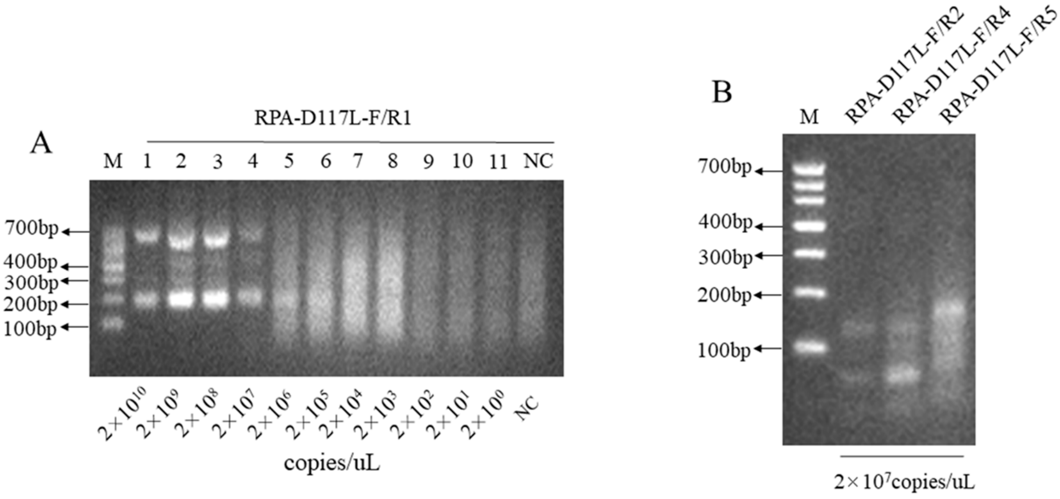

Figure S8. The RPA amplification results with different primers. **A**, RPA with primer pair 1 by using different concentrations of pCold-I-D117L. **B**, RPA with primer pairs 2, 4 and 5. NC denotes negative control.
