# Supplementary material for: Rapid Visual Detection of African Swine Fever Virus with a CRISPR/Cas12a Lateral Flow Strip Based on Structural Protein Gene D117L
Source: Animals (Basel). 2023 Nov 30;13(23):3712. doi: 10.3390/ani13233712 (PMC10705096; doi:10.3390/ani13233712)
Supplement: Supplementary file 1 [file animals-13-03712-s001.zip › Tables S1.pdf]

Table S1. Primers used in this study

Primer names	Primer sequences（5’ - 3’）
pET28a-Cas12a-F	GTGCCGCGCGGCAGCCATATGATGAGCAAGCTGGAGAAGTTT
pET28a-Cas12a-R	GTGGTGGTGGTGCTCGAGTTAGTGCTTCACGCTGGTCTGGGC
T1-F	GCTTGTGGCCGTTTACGTCGCCGTCCAGCTCGACCAGGATGGGCACCACCCCGGC
T1-R	GCCGGGGTGGTGCCCATCCTGGTCGAGCTGGACGGCGACGTAAACGGCCACAAGC
T1-crRNA1-F	GAAATTAATACGACTCACTATAGGGTAATTTCTACTAAGTGTAGATCGTCGCCGTCCAGCTCGACC
T1-crRNA1-R	GGTCGAGCTGGACGGCGACGATCTACACTTAGTAGAAATTACCCTATAGTGAGTCGTATTAATTTC
D117L-crRNA1-F	GAAATTAATACGACTCACTATAGGAATTTCTACTAAGTGTAGATGGTATGCTCGACTTGCAGTC
D117L-crRNA1-R	GACTGCAAGTCGAGCATACCATCTACACTTAGTAGAAATTCCTATAGTGTCGTCGTATTAATTTC
D117L-crRNA2-F	GAAATTAATACGACTCACTATAGGAATTTCTACTAAGTGTAGATTCATAACCTGTCAACCCGCG
D117L-crRNA2-R	CGCGGGTTGACAGGTTATGAATCTACACTTAGTAGAAATTCCTATAGTGAGTCGTATTAATTTC
Non-target crRNA1-F	GAAATTAATACGACTCACTATAGGAATTTCTACTAAGTGTAGTCATAACCTGTCAACCCGCG
Non-target crRNA1-R	AGTATTGGACAGTTGGGCGCCTACACTTAGTAGAAATTCCTATAGTGTCGTCGTATTAATTTC

RPA-D117L-F1	TTAGCCCATACAATCGCCAAATATCCCGGAACAA
RPA-D117L-R1	TTCTAAAGAATACCGGGAAATGGTGGTGAGGCTC
RPA-D117L-F2	ACAACTGCGATTCTCCTGGGCATTTTGATTTT
RPA-D117L-R2	GTGGTGAGGCTCAGGTTGTTGTACATAGTAGC
RPA-D117L-F3	ACAACTGCGATTCTCCTGGGCATTTTGATTTTGC
RPA-D117L-R3	GTGGTGAGGCTCAGGTTGTTGTACATAGTAGCTA
RPA-D117L-F4	ATCCCGGAACAACTGCGATTCTCCTGGGCATTT
RPA-D117L-R4	GCTCAGGTTGTTGTACATAGTAGCTAGGAGGA
RPA-D117L-F5	ATCCCGGAACAACTGCGATTCTCCTGGGCATT
RPA-D117L-R5	TTCTAAAGAATACCGGGAAATGGTGGTGAGGC
PRRSV-UF	GCCCCTGCCCAYCACG
PRRSV-UR	TCGCCCTAATTGAATAGGTGA
PEDV-N-F1	GGGCCGTCGACATGGCTTCTGTCAGTTTTCAGGATCG
PEDV-N-F1	GGGCCGATATTCATTTCCTGTATCGAAGATCTCGTTGATAATTTCAAC
PKV-R1	GCATGAGTCTATTCTACACAGAGC
PKV-F1	TGTATTTGGCAACGAGACGTATG
PPV4-F1	CTTTGCTTTGTCCAACGCAGA
PPV4-R1	TAGATGTCCTGGCACAGATACTTGAC
PRV-gC-F1	CGAGACCGAGGGCGTCTACAC
PRV-gC-R1	GCCCATCATCAGCGCCTGC
CSFV-UF	CTGGGTGGTCTAAGTCCTGAGTA

CSFV-UR	GATTCAACTCCATGTGCCATGTA
PCV2-F1	CACATCGAGAAAGCGAAAGGAAC
PCV2-R1	TGCGGGCCAAAAAAGGTACAGTT
PCV3-F1	CTGTTATTTTGGATGATTTTTATG
PCV3-R1	CACAGCCGTTACTTCACCC
SIV-HA-F1	AAACCAATGATAGGGCCAA
SIV-HA-R1	TTGCACTACACAGTTACCAC
p72-UF	TGCTCATGGTATCAATCTTATCG
p72-UR	CCACTGGGTTGGTATTCCTC
p72-TaqMan	5′ FAM-TTCCATCAAAGTTCTGCAGCTCTT-TAMRA 3′
